# Poly[tri-μ_2_-aqua-(μ_3_-pyridine-2,4-dicarboxyl­ato-κ^4^ *N*,*O* ^2^:*O* ^2^:*O* ^2′^)barium]

**DOI:** 10.1107/S1600536811020204

**Published:** 2011-06-04

**Authors:** Hoda Pasdar, Shadi Siabi, Behrouz Notash, Hossein Aghabozorg, Naser Foroughifar

**Affiliations:** a Department of Chemistry, North Tehran Branch, Islamic Azad University, Tehran, Iran; b Department of Chemistry, Shahid Beheshti University, G. C. Evin, Tehran 1983963113, Iran

## Abstract

In the polymeric title compound, [Ba(C_7_H_3_NO_4_)(H_2_O)_3_]_*n*_, the Ba^II^ ion is ten-coordinated in an NO_9_ environment by one N atom and three O atoms from three pyridine-2,4-dicarboxyl­ate (pydc) ligands and six water mol­ecules. The μ_3_-pydc ligands and the bridging water mol­ecules connect the Ba atoms into a layer parallel to (100). The crystal packing is stabilized by O—H⋯O and C—H⋯O hydrogen bonds.

## Related literature

For related compounds with pyridine dicarb­oxy­lic acid deriv­atives, see: Aghabozorg *et al.* (2008 ▶, 2011*a* ▶,*b* ▶,*c* ▶,*d* ▶); Noro *et al.* (2005 ▶); Pasdar *et al.* (2011*a* ▶,*b* ▶); Wang *et al.* (2007 ▶).
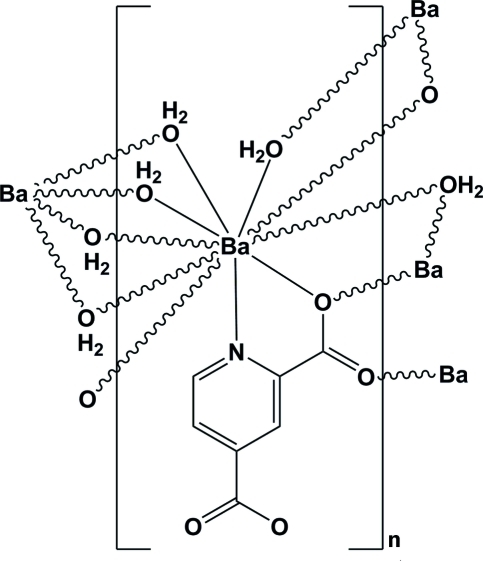


## Experimental

### 

#### Crystal data

[Ba(C_7_H_3_NO_4_)(H_2_O)_3_]
*M* *_r_* = 356.48
Monoclinic, 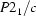

*a* = 11.079 (2) Å
*b* = 13.714 (3) Å
*c* = 6.5961 (13) Å
β = 94.13 (3)°
*V* = 999.6 (3) Å^3^
*Z* = 4
Mo *K*α radiation
μ = 4.00 mm^−1^
*T* = 298 K
0.39 × 0.38 × 0.33 mm

#### Data collection

Stoe IPDS-2T diffractometer
Absorption correction: numerical (*X-SHAPE* and *X-RED32*; Stoe & Cie, 2005 ▶) *T*_min_ = 0.410, *T*_max_ = 0.460
7321 measured reflections
2681 independent reflections
2515 reflections with *I* > 2σ(*I*)
*R*_int_ = 0.041

#### Refinement

*R*[*F* ^2^ > 2σ(*F* ^2^)] = 0.023
*wR*(*F* ^2^) = 0.057
*S* = 1.10
2681 reflections
170 parameters
1 restraint
H atoms treated by a mixture of independent and constrained refinement
Δρ_max_ = 2.20 e Å^−3^
Δρ_min_ = −0.60 e Å^−3^

### 

Data collection: *X-AREA* (Stoe & Cie, 2005 ▶); cell refinement: *X-AREA*; data reduction: *X-AREA*; program(s) used to solve structure: *SHELXS97* (Sheldrick, 2008 ▶); program(s) used to refine structure: *SHELXL97* (Sheldrick, 2008 ▶); molecular graphics: *ORTEP-3* (Farrugia, 1997 ▶); software used to prepare material for publication: *WinGX* (Farrugia, 1999 ▶).

## Supplementary Material

Crystal structure: contains datablock(s) I, global. DOI: 10.1107/S1600536811020204/hy2431sup1.cif

Structure factors: contains datablock(s) I. DOI: 10.1107/S1600536811020204/hy2431Isup2.hkl

Additional supplementary materials:  crystallographic information; 3D view; checkCIF report

## Figures and Tables

**Table 1:** Hydrogen-bond geometry (Å, °)

*D*—H⋯*A*	*D*—H	H⋯*A*	*D*⋯*A*	*D*—H⋯*A*
C5—H5⋯O2^i^	0.93	2.48	3.161 (3)	130
O5—H5*A*⋯O1^ii^	0.77 (5)	2.14 (4)	2.881 (3)	162 (4)
O5—H5*B*⋯O4^iii^	0.77 (5)	2.02 (5)	2.785 (3)	174 (4)
O6—H6*A*⋯O4^iv^	0.91 (4)	2.03 (4)	2.865 (3)	151 (3)
O6—H6*B*⋯O3^v^	0.76 (4)	2.08 (4)	2.816 (3)	167 (4)
O7—H7*A*⋯O3^v^	0.85 (5)	1.96 (5)	2.809 (3)	175 (4)
O7—H7*B*⋯O4^vi^	0.75 (4)	2.11 (4)	2.810 (3)	155 (4)

Symmetry codes: (i) 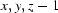; (ii) 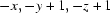; (iii) 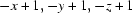; (iv) 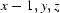; (v) 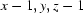; (vi) 
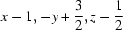
.

**Fig. 1.**
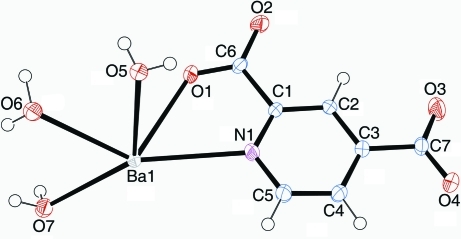
The asymmetric unit of the title compound, with displacement ellipsoids drawn at 50% probability level.

**Fig. 2.**
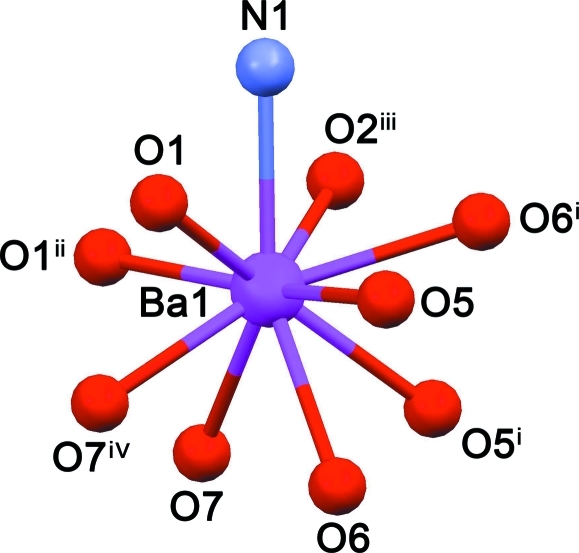
The coordination environment around BaII ion in the title compound. [Symmetry codes: (i) -x, -y+1, -z; (ii) x, -y+3/2, z-1/2; (iii) x, y, z-1; (iv) x, -y+3/2, z+1/2.]

**Fig. 3.**
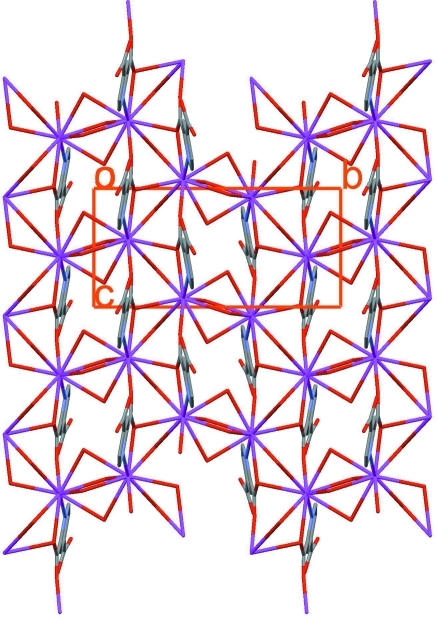
A view of the two-dimensional structure of the title compound viewed down the a axis. H atoms have been omitted for clarity.

## supplementary crystallographic information

### Comment

Pyridine dicarboxylic acid derivatives, depending on the composition and situation of carboxylic groups and the number of deprotonated carboxylic groups, can form wide variety of compounds from organic proton transfer compounds (Aghabozorg *et al.*, 2011*a*,*c*,*d*) to discrete coordination compounds (Aghabozorg *et al.*, 2008, 2011*b*; Noro *et al.*, 2005; Pasdar *et al.*, 2011*a*) and coordination polymers (Pasdar *et al.*, 2011*b*).

The asymmetric unit of the title compound is shown in Fig. 1. Two carboxylate groups of the pyridine-2,4-dicarboxylate (pydc) ligand are deprotonated and Ba^II^ ion is ten-coordinated in an NO_9_ environment (Fig. 2). The crystal structure shows that the compound is a two-dimensional polymer (Fig. 3). O—H···O and C—H···O hydrogen bonds stabilize the crystal packing (Table 1).

### Experimental

A mixture of Ba(NO_3_)_2_ (0.132 g), pyridine-2,4-dicarboxylic acid (0.085 g), 2,2'-bipyridine (0.156 g) in H_2_O (60 ml) was stirred at 40°C for 1 h. The solution was filtered, and the filtrate was stand at room temperature. After two weaks, colorless block-shaped crystals of the title compound were obtained.

### Refinement

H atoms of water molecules were found in a difference Fourier map and refined isotropically. H6B was refined with a distance restraint of O—H = 0.75 (3). C-bound H atoms were positioned geometrically and refined as riding atoms, with C—H = 0.93 Å and *U*_iso_(H) = 1.2*U*_eq_(C). The highest residual electron density was found at 0.80 Å from Ba1 atom and the deepest hole at 0.80 Å from Ba1 atom.

### Crystal data

**Table:**

[Ba(C_7_H_3_NO_4_)(H_2_O)_3_]	*F*(000) = 680
*M**_r_* = 356.48	*D*_x_ = 2.369 Mg m^−^^3^
Monoclinic, *P*2_1_/*c*	Mo *K*α radiation, λ = 0.71073 Å
Hall symbol: -P 2ybc	Cell parameters from 2681 reflections
*a* = 11.079 (2) Å	θ = 2.4–29.1°
*b* = 13.714 (3) Å	µ = 4.00 mm^−^^1^
*c* = 6.5961 (13) Å	*T* = 298 K
β = 94.13 (3)°	Block, colorless
*V* = 999.6 (3) Å^3^	0.39 × 0.38 × 0.33 mm
*Z* = 4	

### Data collection

**Table:**

Stoe IPDS-2T diffractometer	2681 independent reflections
Radiation source: fine-focus sealed tube	2515 reflections with *I* > 2σ(*I*)
graphite	*R*_int_ = 0.041
ω scans	θ_max_ = 29.1°, θ_min_ = 2.4°
Absorption correction: numerical (*X-SHAPE* and *X-RED32*; Stoe & Cie, 2005)	*h* = −15→13
*T*_min_ = 0.410, *T*_max_ = 0.460	*k* = −18→18
7321 measured reflections	*l* = −9→9

### Refinement

**Table:**

Refinement on *F*^2^	Secondary atom site location: difference Fourier map
Least-squares matrix: full	Hydrogen site location: inferred from neighbouring sites
*R*[*F*^2^ > 2σ(*F*^2^)] = 0.023	H atoms treated by a mixture of independent and constrained refinement
*wR*(*F*^2^) = 0.057	*w* = 1/[σ^2^(*F*_o_^2^) + (0.0343*P*)^2^ + 0.2871*P*] where *P* = (*F*_o_^2^ + 2*F*_c_^2^)/3
*S* = 1.10	(Δ/σ)_max_ = 0.002
2681 reflections	Δρ_max_ = 2.20 e Å^−^^3^
170 parameters	Δρ_min_ = −0.60 e Å^−^^3^
1 restraint	Extinction correction: *SHELXL97* (Sheldrick, 2008), Fc^*^=kFc[1+0.001xFc^2^λ^3^/sin(2θ)]^-1/4^
Primary atom site location: structure-invariant direct methods	Extinction coefficient: 0.0182 (7)

### Fractional atomic coordinates and isotropic or equivalent isotropic displacement parameters (Å^2^)

**Table:**

	*x*	*y*	*z*	*U*_iso_*/*U*_eq_	
H6B	−0.211 (3)	0.577 (3)	−0.019 (6)	0.045 (11)*	
O6	−0.16582 (16)	0.54989 (14)	0.0517 (3)	0.0245 (3)	
O7	−0.09028 (16)	0.75423 (14)	−0.1846 (3)	0.0234 (3)	
O5	0.02949 (17)	0.45857 (14)	0.2957 (3)	0.0233 (3)	
Ba1	0.074526 (10)	0.638147 (8)	0.073630 (17)	0.01506 (7)	
C2	0.4566 (2)	0.63768 (15)	0.5688 (4)	0.0179 (4)	
H2	0.4672	0.6508	0.7073	0.021*	
N1	0.32012 (18)	0.61649 (17)	0.2709 (3)	0.0223 (4)	
C1	0.3411 (2)	0.63417 (14)	0.4713 (4)	0.0166 (4)	
C5	0.4167 (2)	0.6023 (2)	0.1646 (4)	0.0290 (5)	
H5	0.4040	0.5907	0.0258	0.035*	
O1	0.13032 (15)	0.66224 (14)	0.4870 (3)	0.0227 (3)	
O2	0.24279 (19)	0.65068 (17)	0.7766 (3)	0.0341 (5)	
C6	0.2301 (2)	0.64984 (16)	0.5894 (4)	0.0175 (4)	
C7	0.6829 (2)	0.62030 (18)	0.5578 (4)	0.0206 (4)	
C3	0.5562 (2)	0.62132 (17)	0.4560 (4)	0.0186 (4)	
C4	0.5348 (2)	0.6037 (2)	0.2492 (4)	0.0268 (5)	
H4	0.5990	0.5930	0.1685	0.032*	
O3	0.69566 (19)	0.64657 (16)	0.7382 (3)	0.0340 (5)	
O4	0.76764 (15)	0.59321 (15)	0.4537 (3)	0.0278 (4)	
H5A	−0.019 (4)	0.438 (3)	0.361 (6)	0.047 (11)*	
H5B	0.087 (4)	0.448 (3)	0.364 (6)	0.048 (12)*	
H7B	−0.116 (4)	0.792 (3)	−0.116 (6)	0.039 (10)*	
H6A	−0.209 (4)	0.550 (3)	0.164 (6)	0.043 (10)*	
H7A	−0.155 (4)	0.723 (3)	−0.215 (7)	0.060 (13)*	

### Atomic displacement parameters (Å^2^)

**Table:**

	*U*^11^	*U*^22^	*U*^33^	*U*^12^	*U*^13^	*U*^23^
O6	0.0208 (8)	0.0317 (9)	0.0211 (8)	0.0043 (7)	0.0026 (7)	0.0017 (7)
O7	0.0204 (8)	0.0251 (8)	0.0246 (8)	−0.0010 (7)	0.0016 (7)	−0.0014 (7)
O5	0.0192 (8)	0.0316 (9)	0.0191 (8)	0.0005 (7)	0.0014 (7)	0.0040 (7)
Ba1	0.01347 (9)	0.01844 (9)	0.01335 (10)	0.00009 (4)	0.00141 (5)	−0.00001 (4)
C2	0.0153 (10)	0.0255 (11)	0.0128 (10)	−0.0002 (7)	0.0004 (8)	0.0007 (7)
N1	0.0169 (9)	0.0350 (10)	0.0148 (9)	0.0023 (8)	−0.0012 (7)	−0.0034 (8)
C1	0.0128 (9)	0.0217 (10)	0.0156 (10)	0.0007 (7)	0.0026 (8)	0.0003 (7)
C5	0.0177 (10)	0.0535 (16)	0.0157 (10)	0.0048 (11)	0.0003 (8)	−0.0053 (11)
O1	0.0135 (7)	0.0330 (8)	0.0218 (8)	0.0041 (6)	0.0017 (6)	0.0010 (7)
O2	0.0216 (9)	0.0644 (14)	0.0170 (9)	−0.0006 (8)	0.0052 (7)	−0.0048 (8)
C6	0.0138 (9)	0.0202 (9)	0.0189 (10)	−0.0006 (7)	0.0042 (8)	−0.0010 (8)
C7	0.0137 (9)	0.0240 (9)	0.0237 (11)	−0.0005 (8)	−0.0013 (8)	0.0044 (9)
C3	0.0146 (9)	0.0227 (9)	0.0182 (10)	0.0006 (8)	0.0002 (8)	0.0014 (8)
C4	0.0160 (10)	0.0466 (15)	0.0179 (11)	0.0017 (10)	0.0026 (8)	−0.0032 (10)
O3	0.0237 (10)	0.0518 (12)	0.0252 (10)	0.0041 (8)	−0.0065 (8)	−0.0064 (8)
O4	0.0156 (7)	0.0406 (10)	0.0272 (9)	0.0030 (7)	0.0023 (6)	0.0028 (8)

### Geometric parameters (Å, °)

**Table:**

O6—H6B	0.75 (3)	C2—C1	1.391 (3)
O6—H6A	0.91 (4)	C2—C3	1.393 (3)
O7—H7B	0.75 (4)	C2—H2	0.9300
O7—H7A	0.85 (5)	N1—C5	1.336 (3)
O5—H5A	0.77 (5)	N1—C1	1.347 (3)
O5—H5B	0.77 (5)	C1—C6	1.518 (3)
Ba1—O1	2.7720 (19)	C5—C4	1.385 (3)
Ba1—O2^i^	2.805 (2)	C5—H5	0.9300
Ba1—O1^ii^	2.8728 (19)	O1—C6	1.265 (3)
Ba1—O7^iii^	2.9111 (18)	O2—C6	1.233 (3)
Ba1—O7	2.8846 (19)	C7—O3	1.242 (3)
Ba1—O6^iv^	2.9115 (19)	C7—O4	1.258 (3)
Ba1—O5^iv^	2.935 (2)	C7—C3	1.511 (3)
Ba1—O5	2.9263 (19)	C3—C4	1.389 (3)
Ba1—N1	2.945 (2)	C4—H4	0.9300
Ba1—O6	2.9190 (19)		
			
Ba1^iv^—O6—Ba1	92.69 (5)	O7—Ba1—N1	147.33 (6)
Ba1^iv^—O6—H6B	119 (3)	O7^iii^—Ba1—N1	113.88 (6)
Ba1—O6—H6B	113 (3)	O6^iv^—Ba1—N1	72.99 (6)
Ba1^iv^—O6—H6A	117 (2)	O6—Ba1—N1	142.22 (6)
Ba1—O6—H6A	119 (2)	O5—Ba1—N1	83.07 (6)
H6B—O6—H6A	98 (4)	O5^iv^—Ba1—N1	128.03 (6)
Ba1—O7—Ba1^ii^	102.01 (6)	O1—Ba1—C6^ii^	77.88 (6)
Ba1—O7—H7B	106 (3)	O2^i^—Ba1—C6^ii^	66.76 (6)
Ba1^ii^—O7—H7B	105 (3)	O1^ii^—Ba1—C6^ii^	21.45 (5)
Ba1—O7—H7A	110 (3)	O7—Ba1—C6^ii^	80.97 (6)
Ba1^ii^—O7—H7A	133 (3)	O7^iii^—Ba1—C6^ii^	83.42 (5)
H7B—O7—H7A	98 (4)	O6^iv^—Ba1—C6^ii^	125.75 (5)
Ba1—O5—Ba1^iv^	92.06 (5)	O6—Ba1—C6^ii^	145.09 (5)
Ba1—O5—H5A	138 (3)	O5—Ba1—C6^ii^	144.49 (6)
Ba1^iv^—O5—H5A	93 (3)	O5^iv^—Ba1—C6^ii^	125.70 (5)
Ba1—O5—H5B	107 (3)	N1—Ba1—C6^ii^	67.67 (6)
Ba1^iv^—O5—H5B	131 (3)	O1—Ba1—Ba1^iv^	112.92 (4)
H5A—O5—H5B	101 (4)	O2^i^—Ba1—Ba1^iv^	99.38 (5)
O1—Ba1—O2^i^	124.57 (6)	O1^ii^—Ba1—Ba1^iv^	153.68 (4)
O1—Ba1—O1^ii^	92.64 (5)	O7—Ba1—Ba1^iv^	98.00 (4)
O2^i^—Ba1—O1^ii^	68.86 (6)	O7^iii^—Ba1—Ba1^iv^	109.33 (4)
O1—Ba1—O7	127.00 (5)	O6^iv^—Ba1—Ba1^iv^	43.72 (4)
O2^i^—Ba1—O7	88.74 (6)	O6—Ba1—Ba1^iv^	43.58 (4)
O1^ii^—Ba1—O7	59.52 (5)	O5—Ba1—Ba1^iv^	44.06 (4)
O1—Ba1—O7^iii^	60.32 (5)	O5^iv^—Ba1—Ba1^iv^	43.89 (4)
O2^i^—Ba1—O7^iii^	145.70 (6)	N1—Ba1—Ba1^iv^	110.18 (5)
O1^ii^—Ba1—O7^iii^	77.19 (5)	C1—C2—C3	119.0 (2)
O7—Ba1—O7^iii^	69.42 (3)	C1—C2—H2	120.5
O1—Ba1—O6^iv^	109.12 (5)	C3—C2—H2	120.5
O2^i^—Ba1—O6^iv^	66.05 (6)	C5—N1—C1	116.9 (2)
O1^ii^—Ba1—O6^iv^	134.70 (5)	C5—N1—Ba1	122.17 (16)
O7—Ba1—O6^iv^	122.63 (6)	C1—N1—Ba1	120.28 (14)
O7^iii^—Ba1—O6^iv^	148.11 (5)	N1—C1—C2	123.3 (2)
O1—Ba1—O6	103.66 (6)	N1—C1—C6	116.1 (2)
O2^i^—Ba1—O6	129.84 (6)	C2—C1—C6	120.7 (2)
O1^ii^—Ba1—O6	126.43 (5)	N1—C5—C4	123.8 (2)
O7—Ba1—O6	70.32 (5)	N1—C5—H5	118.1
O7^iii^—Ba1—O6	68.38 (5)	C4—C5—H5	118.1
O6^iv^—Ba1—O6	87.31 (5)	C6—O1—Ba1	129.32 (14)
O1—Ba1—O5	69.02 (5)	C6—O1—Ba1^iii^	102.37 (14)
O2^i^—Ba1—O5	123.15 (6)	Ba1—O1—Ba1^iii^	105.86 (6)
O1^ii^—Ba1—O5	161.49 (5)	C6—O2—Ba1^v^	131.70 (16)
O7—Ba1—O5	129.53 (5)	O2—C6—O1	124.5 (2)
O7^iii^—Ba1—O5	90.93 (5)	O2—C6—C1	118.5 (2)
O6^iv^—Ba1—O5	58.13 (5)	O1—C6—C1	117.0 (2)
O6—Ba1—O5	59.20 (6)	O2—C6—Ba1^iii^	92.52 (15)
O1—Ba1—O5^iv^	156.43 (5)	O1—C6—Ba1^iii^	56.18 (12)
O2^i^—Ba1—O5^iv^	71.89 (6)	C1—C6—Ba1^iii^	122.45 (13)
O1^ii^—Ba1—O5^iv^	110.05 (5)	O3—C7—O4	124.8 (2)
O7—Ba1—O5^iv^	64.24 (6)	O3—C7—C3	117.5 (2)
O7^iii^—Ba1—O5^iv^	117.35 (5)	O4—C7—C3	117.8 (2)
O6^iv^—Ba1—O5^iv^	59.19 (5)	C4—C3—C2	117.8 (2)
O6—Ba1—O5^iv^	57.95 (5)	C4—C3—C7	121.5 (2)
O5—Ba1—O5^iv^	87.94 (5)	C2—C3—C7	120.7 (2)
O1—Ba1—N1	56.21 (6)	C5—C4—C3	119.2 (2)
O2^i^—Ba1—N1	71.07 (6)	C5—C4—H4	120.4
O1^ii^—Ba1—N1	88.79 (6)	C3—C4—H4	120.4
			
Ba1^ii^—O7—Ba1—O1	108.60 (7)	C5—N1—C1—C2	−0.2 (4)
Ba1^ii^—O7—Ba1—O2^i^	−25.36 (7)	Ba1—N1—C1—C2	−171.43 (15)
Ba1^ii^—O7—Ba1—O1^ii^	40.72 (5)	C5—N1—C1—C6	−179.8 (2)
Ba1^ii^—O7—Ba1—O7^iii^	127.63 (8)	Ba1—N1—C1—C6	9.0 (3)
Ba1^ii^—O7—Ba1—O6^iv^	−85.61 (7)	C3—C2—C1—N1	−0.6 (3)
Ba1^ii^—O7—Ba1—O6	−158.86 (7)	C3—C2—C1—C6	178.93 (19)
Ba1^ii^—O7—Ba1—O5	−159.07 (5)	C1—N1—C5—C4	0.7 (4)
Ba1^ii^—O7—Ba1—O5^iv^	−95.80 (7)	Ba1—N1—C5—C4	171.8 (2)
Ba1^ii^—O7—Ba1—N1	25.18 (13)	O2^i^—Ba1—O1—C6	−24.9 (2)
Ba1^ii^—O7—Ba1—C6^ii^	41.31 (6)	O1^ii^—Ba1—O1—C6	−90.95 (18)
Ba1^ii^—O7—Ba1—Ba1^iv^	−124.66 (5)	O7—Ba1—O1—C6	−144.00 (18)
Ba1^iv^—O6—Ba1—O1	−109.03 (5)	O7^iii^—Ba1—O1—C6	−164.6 (2)
Ba1^iv^—O6—Ba1—O2^i^	55.35 (9)	O6^iv^—Ba1—O1—C6	48.6 (2)
Ba1^iv^—O6—Ba1—O1^ii^	147.19 (5)	O6—Ba1—O1—C6	140.52 (19)
Ba1^iv^—O6—Ba1—O7	126.16 (6)	O5—Ba1—O1—C6	91.6 (2)
Ba1^iv^—O6—Ba1—O7^iii^	−158.90 (7)	O5^iv^—Ba1—O1—C6	104.5 (2)
Ba1^iv^—O6—Ba1—O6^iv^	0.0	N1—Ba1—O1—C6	−4.18 (18)
Ba1^iv^—O6—Ba1—O5	−54.04 (5)	C6^ii^—Ba1—O1—C6	−75.3 (2)
Ba1^iv^—O6—Ba1—O5^iv^	54.86 (5)	Ba1^iv^—Ba1—O1—C6	95.48 (19)
Ba1^iv^—O6—Ba1—N1	−57.40 (10)	O2^i^—Ba1—O1—Ba1^iii^	96.43 (7)
Ba1^iv^—O6—Ba1—C6^ii^	162.67 (7)	O1^ii^—Ba1—O1—Ba1^iii^	30.42 (8)
Ba1^iv^—O5—Ba1—O1	174.89 (6)	O7—Ba1—O1—Ba1^iii^	−22.64 (8)
Ba1^iv^—O5—Ba1—O2^i^	−66.73 (8)	O7^iii^—Ba1—O1—Ba1^iii^	−43.21 (5)
Ba1^iv^—O5—Ba1—O1^ii^	166.78 (13)	O6^iv^—Ba1—O1—Ba1^iii^	170.00 (5)
Ba1^iv^—O5—Ba1—O7	53.60 (8)	O6—Ba1—O1—Ba1^iii^	−98.11 (6)
Ba1^iv^—O5—Ba1—O7^iii^	117.34 (5)	O5—Ba1—O1—Ba1^iii^	−147.01 (7)
Ba1^iv^—O5—Ba1—O6^iv^	−54.46 (5)	O5^iv^—Ba1—O1—Ba1^iii^	−134.14 (11)
Ba1^iv^—O5—Ba1—O6	53.36 (6)	N1—Ba1—O1—Ba1^iii^	117.19 (8)
Ba1^iv^—O5—Ba1—O5^iv^	0.0	C6^ii^—Ba1—O1—Ba1^iii^	46.08 (6)
Ba1^iv^—O5—Ba1—N1	−128.71 (6)	Ba1^iv^—Ba1—O1—Ba1^iii^	−143.15 (4)
Ba1^iv^—O5—Ba1—C6^ii^	−162.71 (6)	Ba1^v^—O2—C6—O1	14.9 (4)
O1—Ba1—N1—C5	−174.1 (2)	Ba1^v^—O2—C6—C1	−165.84 (15)
O2^i^—Ba1—N1—C5	−12.0 (2)	Ba1^v^—O2—C6—Ba1^iii^	64.7 (2)
O1^ii^—Ba1—N1—C5	−80.1 (2)	Ba1—O1—C6—O2	−170.54 (18)
O7—Ba1—N1—C5	−66.7 (3)	Ba1^iii^—O1—C6—O2	66.7 (3)
O7^iii^—Ba1—N1—C5	−155.5 (2)	Ba1—O1—C6—C1	10.2 (3)
O6^iv^—Ba1—N1—C5	57.8 (2)	Ba1^iii^—O1—C6—C1	−112.54 (17)
O6—Ba1—N1—C5	119.5 (2)	Ba1—O1—C6—Ba1^iii^	122.77 (18)
O5—Ba1—N1—C5	116.6 (2)	N1—C1—C6—O2	168.6 (2)
O5^iv^—Ba1—N1—C5	34.7 (2)	C2—C1—C6—O2	−11.0 (3)
C6^ii^—Ba1—N1—C5	−84.0 (2)	N1—C1—C6—O1	−12.2 (3)
Ba1^iv^—Ba1—N1—C5	81.3 (2)	C2—C1—C6—O1	168.2 (2)
O1—Ba1—N1—C1	−3.35 (16)	N1—C1—C6—Ba1^iii^	−77.6 (2)
O2^i^—Ba1—N1—C1	158.68 (19)	C2—C1—C6—Ba1^iii^	102.8 (2)
O1^ii^—Ba1—N1—C1	90.65 (18)	C1—C2—C3—C4	1.0 (3)
O7—Ba1—N1—C1	103.99 (18)	C1—C2—C3—C7	−177.42 (19)
O7^iii^—Ba1—N1—C1	15.24 (19)	O3—C7—C3—C4	172.7 (3)
O6^iv^—Ba1—N1—C1	−131.43 (19)	O4—C7—C3—C4	−7.1 (4)
O6—Ba1—N1—C1	−69.8 (2)	O3—C7—C3—C2	−8.9 (3)
O5—Ba1—N1—C1	−72.70 (18)	O4—C7—C3—C2	171.3 (2)
O5^iv^—Ba1—N1—C1	−154.60 (16)	N1—C5—C4—C3	−0.4 (5)
C6^ii^—Ba1—N1—C1	86.74 (18)	C2—C3—C4—C5	−0.5 (4)
Ba1^iv^—Ba1—N1—C1	−108.02 (17)	C7—C3—C4—C5	177.9 (3)

Symmetry codes: (i) *x*, *y*, *z*−1; (ii) *x*, −*y*+3/2, *z*−1/2; (iii) *x*, −*y*+3/2, *z*+1/2; (iv) −*x*, −*y*+1, −*z*; (v) *x*, *y*, *z*+1.

### Hydrogen-bond geometry (Å, °)

**Table:**

*D*—H···*A*	*D*—H	H···*A*	*D*···*A*	*D*—H···*A*
C5—H5···O2^i^	0.93	2.48	3.161 (3)	130
O5—H5A···O1^vi^	0.77 (5)	2.14 (4)	2.881 (3)	162 (4)
O5—H5B···O4^vii^	0.77 (5)	2.02 (5)	2.785 (3)	174 (4)
O6—H6A···O4^viii^	0.91 (4)	2.03 (4)	2.865 (3)	151 (3)
O6—H6B···O3^ix^	0.76 (4)	2.08 (4)	2.816 (3)	167 (4)
O7—H7A···O3^ix^	0.85 (5)	1.96 (5)	2.809 (3)	175 (4)
O7—H7B···O4^x^	0.75 (4)	2.11 (4)	2.810 (3)	155 (4)

Symmetry codes: (i) *x*, *y*, *z*−1; (vi) −*x*, −*y*+1, −*z*+1; (vii) −*x*+1, −*y*+1, −*z*+1; (viii) *x*−1, *y*, *z*; (ix) *x*−1, *y*, *z*−1; (x) *x*−1, −*y*+3/2, *z*−1/2.
